# Intra-Individual Changes in Total Procollagen-Type 1 N-terminal Propeptide in a Korean Adult Population

**DOI:** 10.3390/diagnostics12102399

**Published:** 2022-10-02

**Authors:** Rihwa Choi, Sang Gon Lee, Eun Hee Lee

**Affiliations:** 1 Department of Laboratory Medicine, Green Cross Laboratories, Yongin 16924, Korea; 2 Department of Laboratory Medicine and Genetics, Samsung Medical Center, Sungkyunkwan University School of Medicine, Seoul 06351, Korea; 3 Green Cross Laboratories, Yongin 16924, Korea

**Keywords:** total procollagen-type 1 N-terminal propeptide, P1NP, bone turnover marker, osteoporosis, Korea

## Abstract

We aimed to investigate intra-individual changes in total procollagen-type 1 N-terminal pro-peptide (P1NP), a biochemical marker of bone turnover, to understand patient populations and test utilization in a Korean adult population while considering different definitions of least significant changes by sex, age, and medical institution type. Overall, 31,501 P1NP tests were performed on 24,644 Korean adults (3389 men and 21,255 women) with a median age of 68.9 years (interquartile range, IQR, 61.2–77.2) for osteoporosis evaluation. Among these, 1331 (5.4%) patients (127 men and 1204 women) underwent ≥3 follow-up P1NP measurements. The median follow-up period was 12.5 months (IQR, 11.7–15.9). Among 1331 patients, 64.4% experienced a decrease in P1NP and 35.6% experienced an increase in P1NP during follow-up. Among these, the proportion of patients who experienced serum P1NP changes ≥14.4% from baseline was 92.3%, and the proportion of patients who achieved ≤40 ng/mL (a median level of premenopausal Korean women) during follow-up was 31.8%. The overall proportion of patients that experienced a serum P1NP change exceeding the least significant change during follow-up was not significantly different by the type of medical institution.

## 1. Introduction

The total procollagen-type 1 N-terminal propeptide (P1NP) test is a biochemical marker of bone turnover [1,2]. In osteoporosis, bone turnover markers have an important role in management [3,4,5]. P1NP and the C-terminal telopeptide of type I collagen (β-CTX) in blood have been designated as reference bone turnover markers for bone formation and bone resorption, respectively, by the Joint Committee on Bone Metabolism of the International Osteoporosis Foundation (IOF) and the International Federation of Clinical Chemistry and Laboratory Medicine (IFCC) [3,4,5].

In Korea, the serum P1NP test has been used in clinical laboratories by the new health technology assessment process of the National Evidence-based Healthcare Collaborating Agency as a biomarker for osteoporosis to monitor drug treatment since December 2015. This test has been reimbursed by the Health Insurance Review and Assessment (HIRA) since October 2018. According to the guideline by HIRA, P1NP tests are reimbursable only for specified conditions: (1) the baseline assessment of osteoporosis (before osteoporosis treatment) and (2) a test at three to six months of osteoporosis treatment initiation to monitor drug efficacy. Since August 2019, two follow-up measurements per year are reimbursed in Korea.

Clinical guidelines for osteoporosis recommend the measurement of P1NP as a bone formation marker to monitor anti-osteoporotic medications [3,6,7]. The timing of measurement varies by the type of anti-osteoporotic medications, anabolic or anti-resorptive agent [1,2,3]. The guidelines recommend the measurement of P1NP at one to three months after the initiation of the anabolic agent treatment (abaloparatide, romosozumab, teriparatide, etc.) and at six months after treatment initiation of anti-resorptive agents (bisphosphonate, denosumab, selective estrogen receptor modulators, etc.) [3,6,7]. The term ‘least significant changes’ used for the efficacy threshold for anti-osteoporotic medications represents the smallest difference between sequential laboratory results associated with a true change in the measurement (exceeds the usual variability in untreated people) [7]. In Korea, anti-osteoporotic treatment is considered effective, and the continuation of that treatment is recommended based on the follow-up P1NP level [6,8], when (1) the P1NP level exceeds the least significant change or (2) the P1NP level is within the reference range of premenopausal women [6,8]. During follow-up after treatment, the serum P1NP increased when efficacy was achieved by the anabolic agent treatment, and the serum P1NP decreased when efficacy was achieved with anti-resorptive agents [6,7,8].

Because P1NP has not been historically reimbursed as a biomarker for osteoporosis, this test has not been widely utilized in Korea [4,5,9]. A recent survey performed in Korea revealed that only 25.5% of Korean clinicians use the P1NP test, although the P1NP test is a reference bone formation biomarker recommended by the IOF/IFCC [4,5,9]. In that survey, Korean clinicians have difficulties in the interpretation of the serum P1NP test (a bone turnover marker). Therefore, serum P1NP has been underutilized in Korea. [9].

Furthermore, there are inconsistencies in defining the least significant changes for efficacy threshold after treatment among current clinical guidelines about the use of bone turnover markers including P1NP in osteoporosis treatment [3,6]. For example, least significant changes are defined based on a fixed quantitative threshold >10 ng/mL (=10 µg/L) [6,10], a decrease in P1NP to ≤35 ng/mL [7], a decrease in P1NP to ≤40 ng/mL [6,11], or based on a percentage change from baseline, such as ≥14.4% (defined as 2.77×intra-individual coefficient of variation) [12,13], ≥20% [10,14], ≥30% [11], ≥38% [7,10,14], or ≥40% [10,14]. Information on the intra-individual changes in quantitative serum P1NP level during follow-up after treatment is limited from a large Korean population. In addition, the qualitative prevalence of patients who experienced the least significant changes based on these different definitions after treatment is limited and should be examined in a large Korean population.

Meanwhile, among several analytical methods for serum P1NP level, an automated electrochemiluminescence immunoassay manufactured by Roche to measure total serum P1NP has been used in Korean clinical laboratories [6]. Green Cross Laboratories is one of the largest referral clinical laboratories in Korea, providing various types of clinical test services including serum P1NP to various types of medical institutions in Korea. General information about intra-individual changes in serum P1NP test results may help physicians understand basic knowledge with regard to predictive situations during patient management.

Therefore, in this study, we aimed to investigate the P1NP test results to understand the patient population and test utilization in Korea. We also aimed to investigate intra-individual changes in serum P1NP in consideration of different criteria for least significant changes according to different clinical guidelines for osteoporosis. This study can help clinicians understand the general proportion of patients expected to experience changes in P1NP levels based on different definitions of least significant changes.

## 2. Materials and Methods

### 2.1. Subjects

We retrospectively reviewed data obtained through the laboratory information system of Green Cross Laboratories between 1 October 2019 and 22 September 2021 for Korean adults (age > 20 years) who underwent serum P1NP testing. Among them, results were excluded for patients with missing data for age or sex. In order to investigate intra-individual changes in serum P1NP levels, patients who had ≥3 serum P1NP measurements were included in the analysis. Clinical specimens for the serum P1NP test were obtained from different hospitals throughout Korea and sent to the central referral laboratory, Green Cross Laboratories in Yongin, Korea, where all specimens were analyzed (Green Cross Laboratories in Yongin, Korea). We investigated the quantitative and qualitative results of the P1NP by sex, age group, and type of medical institution (public medical centers, local clinics, referral clinical laboratories, hospitals, and university hospitals). All data were anonymized prior to statistical analysis.

### 2.2. Analytical Methods

Serum P1NP was analyzed using an automated immunoassay using an Elecsys total P1NP reagent kit (Roche, Mannheim, Germany) on an e602 analyzer (Roche, Mannheim, Germany). The analytical measurement range of the serum P1NP assay was 5.0–1200.0 ng/mL.

### 2.3. Definitions

The proportions of patients that experienced changes from baseline in serum P1NP during follow-up by eight different definitions of least significant changes described in different clinical guidelines were investigated as follows: (1) a fixed quantitative threshold >10 ng/mL (=10 µg/L) [6,10], (2) a decrease in P1NP to ≤35 ng/mL [7], (3) a decrease in P1NP to ≤40 ng/mL [6,11], (4) a ≥14.4% change from the baseline level (defined as 2.77 × intra-individual coefficient of variation) [12,13], (5) a ≥20% change from the baseline level [10,14], (6) a ≥30% change from the baseline level [11], (7) a ≥38% change from the baseline level [7,10,14], or (8) a ≥40% change from the baseline level [10,14]. To investigate whether the intra-individual changes exceeded the least significant changes, patients were classified by age as follows: ≤49 years, 50–59 years, 60–69 years, 70–79 years, and ≥80 years. Because clinical information on osteoporosis is limited in Green Cross Laboratories, patients were grouped based on serum P1NP decrease or increase during follow-up (when sufficient efficacy was achieved, serum P1NP was increased after anabolic treatment and decreased after the application of anti-resorptive agents) in order to investigate the differences between patients with different anti-osteoporotic management (indirect approach for treatment with anabolic agents or anti-resorptive agents) [4,6,7,9,10].

### 2.4. Statistical Analysis

A non-parametric test was adopted where appropriate for non-normally distributed continuous variables (age, follow-up numbers, follow-up period, and serum P1NP levels). Chi-square tests were used to compare the categorical variables (sex, age group, decrease or increase in serum P1NP during follow-up, least significant changes, and type of medical institution). Statistical analysis was executed using MedCalc Statistical Software version 20.111 (MedCalc Software bv, Ostend, Belgium; https://www.medcalc.org; 2022, accessed on 18 September 2022). *p* values were considered significant at the 0.05 level.

### 2.5. Ethical Approval

This study was conducted according to the guidelines outlined in the Declaration of Helsinki, and all procedures involving human subjects were approved by the Institutional Review Board of Green Cross Laboratories (GCL-2022-1023-01, 3 May 2022). A waiver of informed consent was approved by the IRB because this study was retrospective and involved no more than minimal risk to subjects.

## 3. Results

### 3.1. Characteristics of Study Subjects

During the study period, 31,501 P1NP tests were performed on 24,644 Korean adults (3389 men and 21,255 women) with a median age of 68.9 years (interquartile range, IQR, 61.2–77.2). Among these, 1331 (5.4%) patients underwent ≥3 follow-up P1NP measurements. The baseline patient characteristics are summarized in Table 1.

The median follow-up period was 12.5 months (IQR, 11.7–15.9). Most patients were post-menopausal women aged ≥60 years (72.1%, 959/1331). Women aged 60–69 years were the most prevalent age group (34.9% of total subjects). Among five types of medical institutions, the largest proportion of patients with measured serum P1NP was from those who visited local clinics (34.7%), followed by public medical centers (25.1%) and university hospitals (20.8%). The median age of patients who visited university hospitals was significantly lower (64.1 years, IQR 56.7 to 71.3) than that for other types of medical institutions (*p* < 0.001).

### 3.2. Serum P1NP Level at Initial Measurement

**Figure 1:**
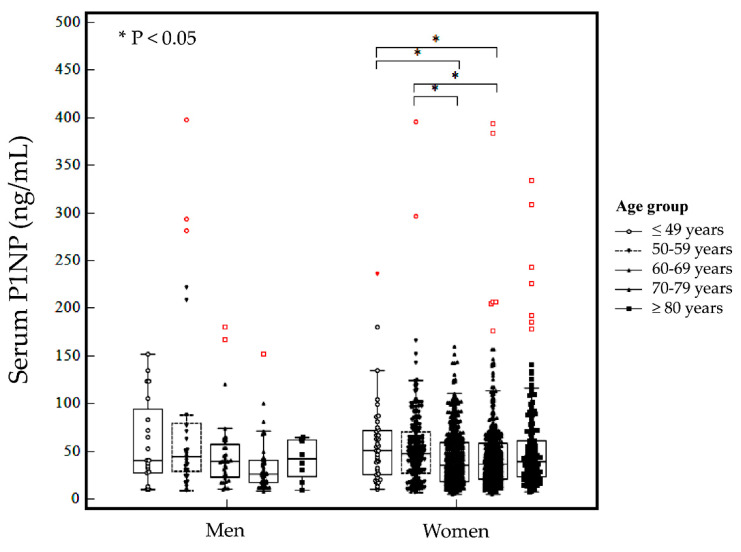
Baseline serum P1NP level by sex and age group. The middle line represents the median, and the central box represents values from the lower to upper quartile (25 to 75th percentiles). Red color represents a value larger than the upper quartile plus 3 times the interquartile range.

**Figure 2:**
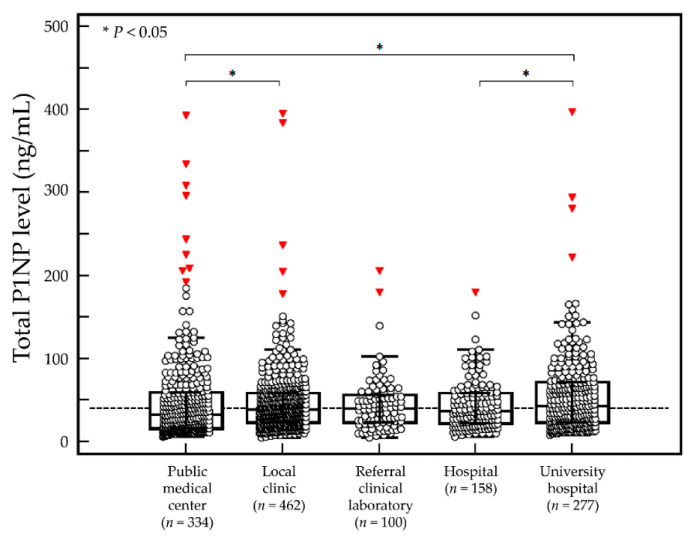
Baseline serum P1NP level during initial measurement by type of medical institution for 1331 Korean adults. The middle line represents the median, and the central box represents values from the lower to upper quartiles (25 to 75th percentiles). (○) represents individual serum P1NP result, and (▼) represents outlier.

The baseline serum P1NP level was not significantly different between men and women (*p* ≥ 0.05). In men, the baseline serum P1NP level was not significantly different among age groups (*p* ≥ 0.05, Figure 1). In women, the baseline serum P1NP level was significantly different between age groups (*p* = 0.002). In post hoc analysis, the serum P1NP level in women aged <59 years was significantly different from that in women aged ≥60 years (*p* < 0.05, except for patients aged ≥80 years). The baseline serum P1NP level was significantly different among patients who visited different types of medical institutions (*p* = 0.004, Figure 2).

### 3.3. Serum P1NP Level during Follow-Up

Among 1331 patients (127 men and 1204 women), 64.4% experienced a decrease in P1NP and 35.6% experienced an increase in P1NP during follow-up. The overall proportion of patients who experienced a decrease or increase in P1NP during follow-up was not significantly different by sex (*p* ≥ 0.05).

**Figure 3:**
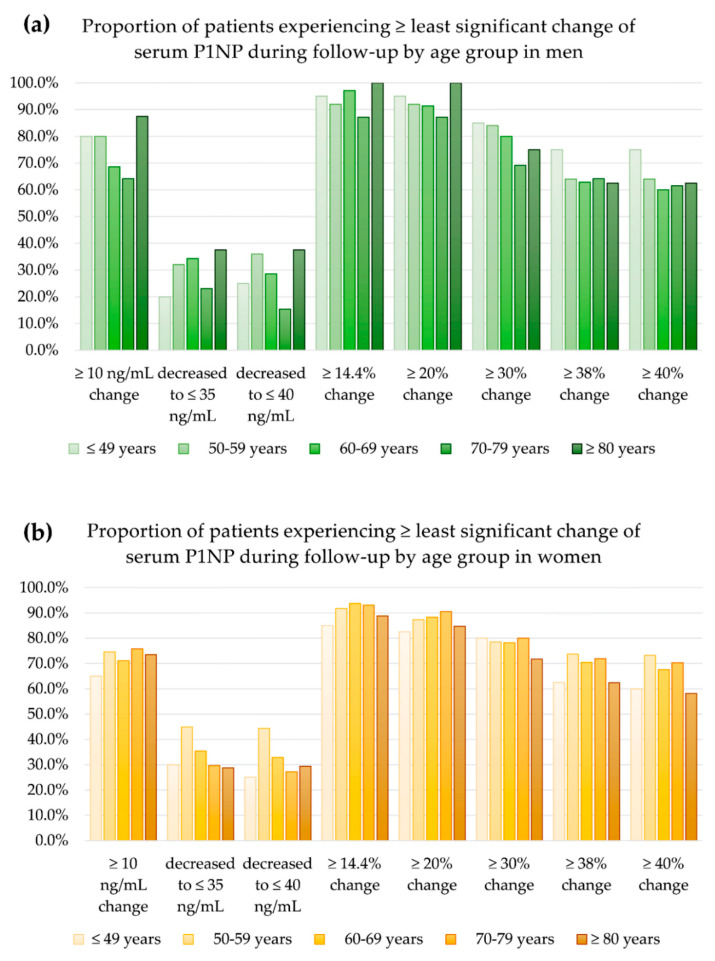
The proportion of patients experiencing changes in serum P1NP exceeding the least significant changes during follow-up: (**a**) in men and (**b**) in women.

**Figure 4:**
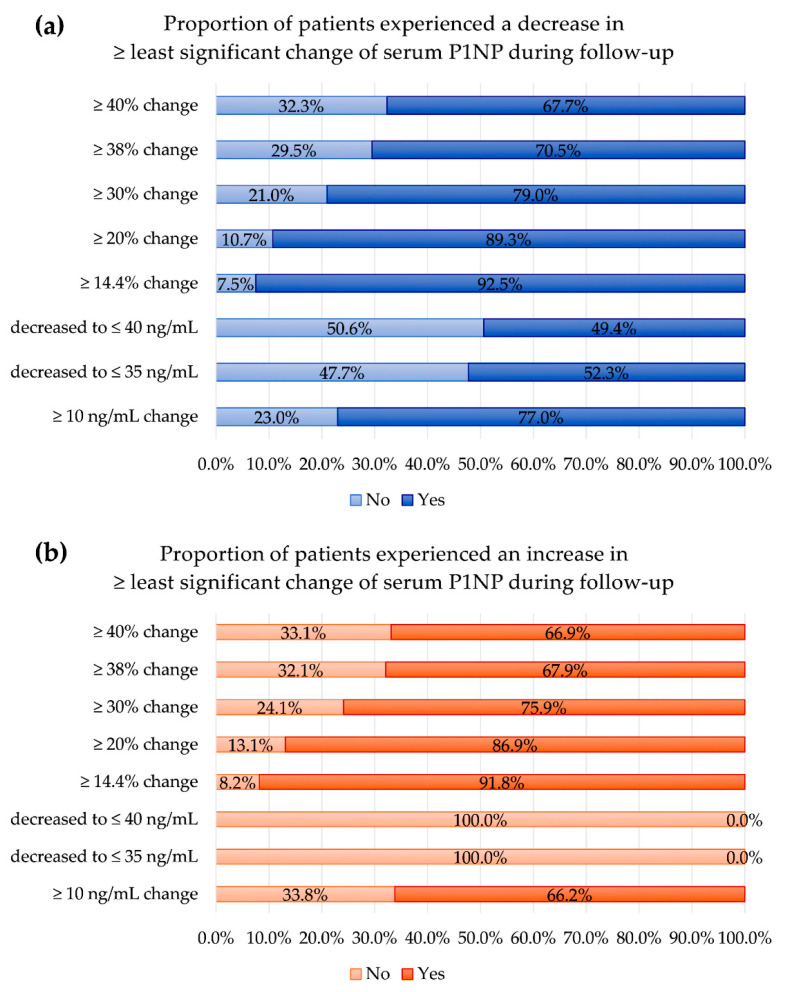
The proportion of patients that experienced changes in serum P1NP exceeding the least significant changes during follow-up: (**a**) in patients experiencing a decrease in serum P1NP and (**b**) in patients experiencing an increase in serum P1NP. ‘No’ represents no achievement of least significant changes, and ‘Yes’ represents an achievement of least significant changes.

The proportions of patients that achieved changes that exceeded the least significant changes by sex, age, and definition are summarized in Figure 3 and Figure 4.

The proportions of patients who achieved changes that exceeded the least significant changes were not significantly different among age groups in men (*p* ≥ 0.05) but did vary significantly in women (*p* < 0.05, Figure 3). The proportion of patients who achieved a decrease in ≤35 ng/mL, *p* = 0.002), a decrease ≤40 ng/mL (*p* < 0.001), and ≥40% changes (*p* = 0.043) during follow-up was significantly different by age group.

The proportion of patients who achieved changes that exceeded the least significant changes for all criteria was not significantly different among types of medical institutions (*p* ≥ 0.05).

Among 857 patients who experienced a decrease in serum P1NP, the largest proportion who achieved changes that exceeded the least significant changes (92.4%) was observed with a definition of least significant change being a ≥14.4% change from the baseline, and the smallest proportion was observed with a definition of a quantitative threshold decrease to ≤40 ng/mL (the median P1NP level of premenopausal Korean women).

Among 474 patients who experienced an increase in serum P1NP, the largest proportion who achieved changes that exceeded the least significant changes (91.8%) was observed when the definition for the least significant change was a ≥14.4% change from the baseline, and the smallest proportion was observed when the definition for the least significant change was a quantitative threshold increase to ≥10.0 ng/mL. A quantitative threshold decrease to ≤ 35 ng/mL or ≤40 ng/mL for the definition for the least significant change was not applicable for patients who experienced an increase in serum P1NP level (0.0%).

### 3.4. Quantitative Changes in Serum P1NP Levels during Follow-Up

**Figure 5:**
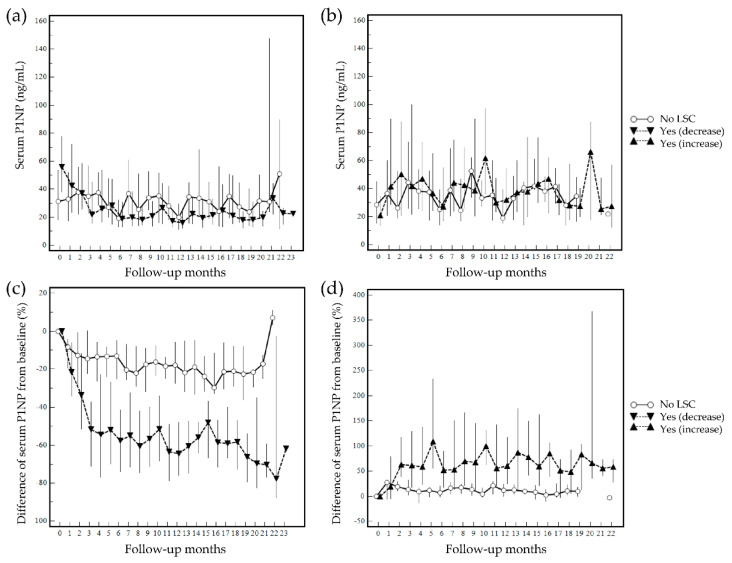
Changes in serum P1NP at follow-up (median serum P1NP level by follow-up month is presented and grouped based on least significant changes (LSC) ≥40% from baseline): (**a**) quantitative changes in patients who experienced a decrease in P1NP during follow-up; (**b**) quantitative changes in patients who experienced an increase in P1NP during follow-up; (**c**) %difference from baseline in patients who experienced a decrease in P1NP during follow-up; (**d**) %difference from baseline in patients who experienced an increase in P1NP during follow-up. ‘No’ represents no achievement of least significant changes, and ‘Yes’ represents an achievement of least significant changes.

**Figure 6:**
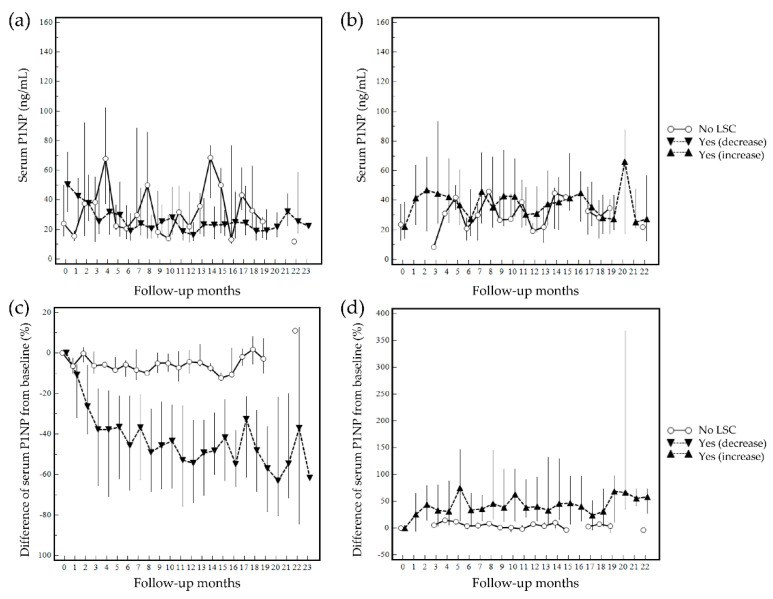
Changes in serum P1NP at follow-up (median serum P1NP level by follow-up month is presented and grouped based on least significant changes (LSC) ≥14.4% from baseline): (**a**) quantitative changes in patients who experienced a decrease in P1NP during follow-up; (**b**) quantitative changes in patients who experienced an increase in P1NP during follow-up; (**c**) %difference from baseline in patients who experienced a decrease in P1NP during follow-up; (**d**) %difference from baseline in patients who experienced an increase in P1NP during follow-up. ‘No’ represents no achievement of least significant changes, and ‘Yes’ represents an achievement of least significant changes.

Quantitative changes in serum P1NP levels during follow-up were investigated. The overall changes in quantitative serum P1NP levels at follow-up measurements (months) based on the least significant changes ≥40% from the baseline are summarized in Figure 5. The overall changes in quantitative serum P1NP levels at follow-up months by the least significant changes ≥14.4% from the baseline are presented in Figure 6. The discrimination between patients who achieved the least significant changes or not was evident when the %difference graphs were used to monitor the quantitative level of serum P1NP instead of the absolute level.

## 4. Discussion

In this study, we retrospectively evaluated serum P1NP test results in Korean adults who visited different types of medical institutions to investigate test utilization and intra-individual changes using different definitions of least significant changes.

Understanding patient populations and test utilization is a practical way to provide general information regarding epidemiologic implications for physicians as well as to provide information regarding predictive values used for the performance evaluation of a specific clinical test [15,16].

In this study, the number of women with tested serum P1NP was larger than that of men, and women aged 60–69 years represented the most prevalent age group. This population distribution was comparable with the proportion of patients managed for osteoporosis in Korea [13]. According to the public database Healthcare Bigdata Hub by HIRA, a total of 8,294,394 patients was managed for osteoporosis in Korea between October 2018 and September 2021, and 35.4% of them were women aged 60–69 years (M81 for osteoporosis, International Classification of Diseases, 10th Revision, http://opendata.hira.or.kr/op/opc/olap3thDsInfo.do, accessed on 20 May 2022). The high prevalence of osteoporosis among post-menopausal women was comparable with previous studies performed in other ethnic populations [1,3,13,17,18,19,20].

Several measurement methods for the serum P1NP assay are available globally, including manual immunoassays manufactured by Orion Diagnostics (Finland) measuring intact P1NP; that of Uscn, Life Science (China) measuring total P1NP; and automated assays manufactured by Immunodiagnostics Systems (IDS, UK) measuring intact P1NP and by Roche (Germany) measuring total P1NP [21,22,23]. Considering that the analytical method for serum P1NP assays used in clinical laboratories in Korea is an automated electrochemiluminescence immunoassay manufactured by Roche, the validation of the analytical methods used in this study will contribute to the generalizability of this study and the standardization of data about biomarkers in osteoporosis treatment in the Korean population [2,8,21,22].

The public database Healthcare Bigdata Hub by HIRA is available for an assessment of the numbers of reimbursed serum P1NP tests and patients in Korea (test code D5030, http://opendata.hira.or.kr/op/opc/olapDiagBhvInfo.do, accessed on 11 May 2022). Ac-cording to the data for the number of patients tested for reimbursed serum P1NP tests be-tween October 2018 and September 2021, the number of patients tested for serum P1NP increased by more than twice from 9130 patients in October 2018 to 22,431 patients in September 2021 (overall, 605,827 patients including repeat visits during a period were tested for serum P1NP). In Green Cross Laboratories, 24,644 patients were tested for serum P1NP during the similar period, with about 4.1% of overall patients tested for serum P1NP in Korea. The large number of patients tested for serum P1NP and test results by sex, age group, and type of medical institution are strengths of this study, which may be representative of the Korean population.

The medical service accessibility is very good in Korea, meaning patients can access local clinics for primary care to tertiary university hospitals easily. Some types of diseases and some types of clinical tests are utilized significantly differently by different types of medical institution according to the severity of medical conditions or reimbursement issues in Korea [24]. In this study, the baseline serum P1NP level varied by sex and age, comparable to findings in previous studies [2,6,9,21,25]. In this study, the median age of patients who visited university hospitals was significantly lower than that for other types of medical institutions, and the baseline serum P1NP level was highest in patients who visited university hospitals and lowest in patients who visited public medical centers. These findings suggest that physicians should understand the differences in serum P1NP levels by sex and age when the serum P1NP level is used in the clinical field [6,7,9,21,25].

In this study, the change in serum P1NP during follow-up was investigated with the assumption that the direction of change (decrease or increase) varies with the type of anti-osteoporotic treatment, because the serum P1NP level is increased after anabolic agent treatment and decreased after anti-resorptive treatment [1,3,7]. In this study, the number of patients who showed decreased serum P1NP levels (64.4%) was greater than the number of patients who showed increased serum P1NP during follow-up (35.6%). This result suggests that more patients might be treated with anti-resorptive agents [6,9]. In this study, the overall proportion of patients who experienced a change (decrease and increase) in P1NP during follow-up was not significantly different by sex.

In this study, the proportion of patients who achieved changes that exceeded the least significant changes for all criteria was not significantly different among types of medical institutions. This suggests that treatment strategies may not significantly differ among physicians in different medical institutions in Korea. In this study, the proportion of patients achieving change regardless of treatment options (only follow-up measurements without treatment or type of anti-osteoporosis therapy) was assessed to provide general information with regard to heterogeneous Korean patient populations. The results of this study expand the baseline knowledge for clinical trials of responses to various types of anti-osteoporotic treatments.

Although serum P1NP measurements are recommended for the use of a reference bone formation biomarker to monitor the therapeutic efficacy of anti-osteoporosis therapies by the IOF/IFCC, the relatively late introduction of this in Korean populations requires additional clarification for its use as a biomarker [6,8,9]. Limited data about serum P1NP changes in Korean populations led to difficulties in the interpretation of test results and the underutilization of this test [8,9]. According to the position statement on the use of bone turnover markers for osteoporosis treatment by the Bone Turnover Marker Committee from the Korean Society for Bone Mineral Research, a sufficient consensus has not been achieved for determining a cut-off point of bone turnover markers that predicts an increased fracture risk or assesses the response to treatment [6].

In this study, data regarding serum P1NP levels during follow-up were presented as both absolute level changes and %differences. Because the %difference from the baseline measurement was more intuitive than the absolute serum P1NP level change, the serum P1NP test results of %difference monitoring with graphical visualization through a laboratory information system can provide the easier interpretation of biomarker response to therapeutic treatment. In addition, such presentation might be helpful for physicians to assess the therapeutic efficacy of anti-osteoporotic treatment. Furthermore, this approach can increase the appropriate utilization of serum P1NP in Korea.

The limitation of this study was a lack of detailed clinical information associated with osteoporosis and various factors that might affect the significance of serum P1NP level, such as type and degree of bone pathologies, history of fracture, radiologic findings, type of anti-osteoporosis therapy, and other medications and comorbidities [14,21]. Because therapeutic efficacies were determined differently by different definitions of least significant changes, physicians should understand the clinical interpretation of serum P1NP changes along with other clinical findings regarding osteoporosis and various factors affecting the serum P1NP level [2,21]. Because this study was a retrospective analysis based on a laboratory information system, detailed clinical information including medical history was not available, and the possibility of other co-founders that might interfere with the measurement was not excluded. Some outliers might affect the distribution of data. The population of this study was heterogeneous, which limits the generalizability of this result. Therefore, caution should be taken during the interpretation of the results. However, this study includes large numbers of study subjects and P1NP test results, and the overall prevalence of the findings has value for epidemiologic insights for physicians, researchers, and laboratory personnel to understand results in routine clinical practice Korea. Future studies based on detailed clinical information are needed to clarify the clinical significance of changes in serum P1NP in Korea.

## 5. Conclusions

In conclusion, we investigated the utilization of the serum P1NP test in Korea to understand patient populations and test utilization in clinical laboratories in a practical, retrospective manner. Intra-individual changes in serum P1NP levels assessed with different criteria for least significant changes may provide basic knowledge with regard to predictive changes in serum P1NP in Korean patient populations. The graphical visualization of serum P1NP level changes as not only absolute concentrations of serum P1NP, but also %differences from baselines during follow-up, is a good option to the improve appropriate utilization of serum P1NP tests. Further studies are needed to investigate the clinical impact of this change in Korean populations.

## Figures and Tables

**Table 1:** Baseline characteristics of 1331 Korean adults.

Characteristics		Total (*n* = 1331)	Men (*n* = 127)	Women (*n* = 1204)
Age, years (median, IQR)		68.1 (60.8 to 76.1)	65.5 (55.9 to 72.6)	68.3 (61.2 to 76.2)
Follow-up measurement, number (median, IQR)		3.0 (3.0 to 3.0)	3.0 (3.0 to 4.0)	3.0 (3.0 to 3.0)
Follow-up duration, months (median, IQR)		12.5 (11.7 to 15.9)	12.1 (9.7 to 14.5)	12.5 (11.8 to 16.2)
P1NP level at initial measurement, ng/mL (median, IQR)		38.5 (20.7 to 61.9)	37.0 (19.9 to 62.1)	38.9 (20.7 to 61.8)
Age distribution (*n*, %)	≤49 years	60 (4.5%)	20 (15.7%)	40 (3.3%)
	50–59 years	230 (17.3%)	25 (19.7%)	205 (17.0%)
	60–69 years	464 (34.9%)	35 (27.6%)	429 (35.6%)
	70–79 years	399 (30.0%)	39 (30.7%)	360 (29.9%)
	≥80 years	178 (13.4%)	8 (6.3%)	170 (14.1%)
Type of medical institution (*n*, %)	Public medical center	334 (25.1%)	25 (19.7%)	309 (25.7%)
	Local clinic	462 (34.7%)	26 (20.5%)	436 (36.2%)
	Referral clinical laboratory	100 (7.5%)	11 (8.7%)	89 (7.4%)
	Hospital	158 (11.9%)	28 (22.0%)	130 (10.8%)
	University hospital	277 (20.8%)	37 (29.1%)	240 (19.9%)
P1NP level by age distribution (median, IQR)	≤49 years	48.8 (26.7 to 73.7)	40.3 (27.5 to 94.3)	50.9 (25.8 to 71.8)
	50–59 years	47.3 (27.0 to 71.4)	44.6 (28.8 to 79.3)	47.5 (27.0 to 70.3)
	60–69 years	36.2 (18.3 to 59.3)	39.3 (22.8 to 57.4)	35.3 (18.1 to 59.4)
	70–79 years	35.1 (20.4 to 56.9)	26.3 (17.3 to 40.7)	36.7 (20.8 to 58.4)
	≥80 years	39.0 (23.4 to 61.2)	42.1 (23.7 to 62.0)	39.0 (23.4 to 61.2)
P1NP level at initial measurement by type of medical institution (*n*, %)	Public medical center	33.2 (16.2 to 59.8)	18.6 (13.3 to 64.4)	33.7 (16.3 to 59.5)
	Local clinic	39.2 (23.6 to 58.9)	37.4 (28.2 to 43.4)	39.7 (23.3 to 59.2)
	Referral clinical laboratory	40.6 (23.6 to 56.9)	46.6 (33.0 to 57.9)	39.6 (22.7 to 56.3)
	Hospital	37.1 (22.3 to 58.3)	35.2 (21.7 to 45.9)	37.6 (22.4 to 58.7)
	University hospital	43.4 (23.3 to 71.9)	40.4 (22.8 to 123.2)	44.3 (23.2 to 70.5)
Increase or decrease in P1NP level during follow-up (*n*, %)	Decrease	857 (64.4%)	76 (59.8%)	781 (64.9%)
	Increase	474 (35.6%)	51 (40.2%)	423 (35.1%)

Abbreviations: P1NP, total procollagen-type 1 N-terminal propeptide; IQR, interquartile range.

## Data Availability

The datasets generated and analyzed during the current study are available from the corresponding authors on reasonable request. The data are not publicly available due to its proprietary nature and ethical concerns.

## References

[1] Bauer D.C. (2019). Clinical Use of Bone Turnover Markers. JAMA.

[2] Szulc P., Naylor K., Hoyle N.R., Eastell R., Leary E.T. (2017). Use of CTX-I and PINP as bone turnover markers: National Bone Health Alliance recommendations to standardize sample handling and patient preparation to reduce pre-analytical variability. Osteoporos. Int..

[3] Watts N.B., Camacho P.M., Lewiecki E.M., Petak S.M. (2021). American Association of Clinical Endocrinologists/American College of Endocrinology Clinical Practice Guidelines for the Diagnosis and Treatment of Postmenopausal Osteoporosis-2020 Update. Endocr. Pract..

[4] Morris H.A., Eastell R., Jorgensen N.R., Cavalier E., Vasikaran S., Chubb S.A.P., Kanis J.A., Cooper C., Makris K. (2017). Clinical usefulness of bone turnover marker concentrations in osteoporosis. Clin. Chim. Acta.

[5] Vasikaran S., Eastell R., Bruyère O., Foldes A.J., Garnero P., Griesmacher A., McClung M., Morris H.A., Silverman S., Trenti T. (2011). Markers of bone turnover for the prediction of fracture risk and monitoring of osteoporosis treatment: A need for international reference standards. Osteoporos. Int..

[6] Park S.Y., Ahn S.H., Yoo J.I., Chung Y.J., Jeon Y.K., Yoon B.H., Kim H.Y., Lee S.H., Lee J., Hong S. (2019). Position Statement on the Use of Bone Turnover Markers for Osteoporosis Treatment. J. Bone Metab..

[7] Eastell R., Szulc P. (2017). Use of bone turnover markers in postmenopausal osteoporosis. Lancet Diabetes Endocrinol.

[8] Park S.Y., Ahn S.H., Yoo J.I., Chung Y.J., Jeon Y.K., Yoon B.H., Kim H.Y., Lee S.H., Lee J., Hong S. (2019). Clinical Application of Bone Turnover Markers in Osteoporosis in Korea. J. Bone Metab..

[9] Ahn S.H., Park S.Y., Yoo J.I., Chung Y.J., Jeon Y.K., Yoon B.H., Kim H.Y., Lee S.H., Lee J., Hong S. (2019). Use of Bone Turnover Markers in Clinical Practice for the Management of Osteoporosis in Korea: From the Survey on the Prescription Pattern of Bone Turnover Markers. J. Bone Metab..

[10] Wu C.H., Chang Y.F., Chen C.H., Lewiecki E.M., Wüster C., Reid I., Tsai K.S., Matsumoto T., Mercado-Asis L.B., Chan D.C. (2021). Consensus Statement on the Use of Bone Turnover Markers for Short-Term Monitoring of Osteoporosis Treatment in the Asia-Pacific Region. J. Clin. Densitom..

[11] The Korean Society for Bone and Mineral Research (2018). Chapter 8, Born turnover biomarkers. Physician’s Guide for Osteoporosis 2018.

[12] Nishizawa Y., Miura M., Ichimura S., Inaba M., Imanishi Y., Shiraki M., Takada J., Chaki O., Hagino H., Fukunaga M. (2019). Executive summary of the Japan Osteoporosis Society Guide for the Use of Bone Turnover Markers in the Diagnosis and Treatment of Osteoporosis (2018 Edition). Clin. Chim. Acta.

[13] Ahn S.H., Park S.M., Park S.Y., Yoo J.I., Jung H.S., Nho J.H., Kim S.H., Lee Y.K., Ha Y.C., Jang S. (2020). Osteoporosis and Osteoporotic Fracture Fact Sheet in Korea. J. Bone Metab..

[14] Diez-Perez A., Naylor K.E., Abrahamsen B., Agnusdei D., Brandi M.L., Cooper C., Dennison E., Eriksen E.F., Gold D.T., Guañabens N. (2017). International Osteoporosis Foundation and European Calcified Tissue Society Working Group. Recommendations for the screening of adherence to oral bisphosphonates. Osteoporos. Int..

[15] CLSI (2008). User Protocol for Evaluation of Qualitative Test Performance; Approved Guideline—Second Edition. CLSI Document EP12A2.

[16] CLSI (2016). Evaluation of Total Analytical Error for Quantitative Medical Laboratory Measurement Procedures. CLSI Guideline EP21, 2nd ed.

[17] Eastell R., O’Neill T.W., Hofbauer L.C., Langdahl B., Reid I.R., Gold D.T., Cummings S.R. (2016). Postmenopausal osteoporosis. Nat. Rev. Dis. Prim..

[18] Black D.M., Rosen C.J. (2016). Clinical Practice. Postmenopausal Osteoporosis. N. Engl. J. Med..

[19] Choi M.H., Yang J.H., Seo J.S., Kim Y.J., Kang S.W. (2021). Prevalence and diagnosis experience of osteoporosis in postmenopausal women over 50: Focusing on socioeconomic factors. PLoS ONE.

[20] Wang L., Yu W., Yin X., Cui L., Tang S., Jiang N., Cui L., Zhao N., Lin Q., Chen L. (2021). Prevalence of Osteoporosis and Fracture in China: The China Osteoporosis Prevalence Study. JAMA Netw. Open.

[21] Bhattoa H.P., Cavalier E., Eastell R., Heijboer A.C., Jørgensen N.R., Makris K., Ulmer C.Z., Kanis J.A., Cooper C., Silverman S.L. (2021). Analytical considerations and plans to standardize or harmonize assays for the reference bone turnover markers PINP and β-CTX in blood. Clin. Chim. Acta.

[22] Cavalier E., Eastell R., Rye Jørgensen N., Makris K., Tournis S., Vasikaran S., Kanis J.A., Cooper C., Pottel H., Morris H.A. (2019). A multicenter study to evaluate harmonization of assays for N-terminal propeptide of type I procollagen (PINP): A report from the IFCC-IOF Joint Committee for Bone Metabolism. Clin. Chem. Lab Med..

[23] Vasikaran S.D., Bhattoa H.P., Eastell R., Heijboer A.C., Jørgensen N.R., Makris K., Ulmer C., Kanis J.A., Cooper C., Silverman S. (2020). Harmonization of commercial assays for PINP; the way forward. Osteoporos. Int..

[24] World Health Organization, Regional Office for the Western Pacific. 2015. Republic of Korea Health System Review. https://apps.who.int/iris/handle/10665/208215.

[25] Yoo J.I., Park A.J., Lim Y.K., Kweon O.J., Choi J.H., Do J.H., Kim S., Kim Y., Ha Y.C. (2018). Age-related Reference Intervals for Total Collagen-I-N-terminal Propeptide in Healthy Korean Population. J. Bone Metab..

